# Competence-Associated Peptide BriC Alters Fatty Acid Biosynthesis in Streptococcus pneumoniae

**DOI:** 10.1128/mSphere.00145-21

**Published:** 2021-05-26

**Authors:** Surya D. Aggarwal, Jessica M. Gullett, Tara Fedder, J. Pedro F. Safi, Charles O. Rock, N. Luisa Hiller

**Affiliations:** a Department of Biological Sciences, Carnegie Mellon University, Pittsburgh, Pennsylvania, USA; b Department of Infectious Diseases, St. Jude Children’s Research Hospital, Memphis, Tennessee, USA; University of Kentucky

**Keywords:** *Streptococcus pneumoniae*, fatty acid biosynthesis, membrane phospholipid composition, biofilms, cell-cell communication, competence

## Abstract

Membrane lipid homeostasis is required for bacteria to survive in a spectrum of host environments. This homeostasis is achieved by regulation of fatty acid chain length and of the ratio of unsaturated to saturated fatty acids. In the pathogen Streptococcus pneumoniae, fatty acid biosynthesis is carried out by a cluster of fatty acid biosynthesis (*fab*) genes (FASII locus) whose expression is controlled by the FabT repressor. Encoded immediately downstream of the FASII locus is BriC, a competence-induced, cell-cell communication peptide that promotes biofilm development as well as nasopharyngeal colonization in a murine model of pneumococcal carriage. Here, we demonstrate that *briC* is cotranscribed with genes of the *fab* gene cluster and that a reduction of *briC* levels, caused by decoupling its transcription from *fab* gene cluster, negatively affects biofilm development. BriC elevates *fabT* transcription, which is predicted to alter the balance of unsaturated and saturated fatty acids produced by the pathway. We find that *briC* inactivation results in a decreased production of unsaturated fatty acids. This affects the membrane properties by decreasing the abundance of di-unsaturated phosphatidylglycerol molecular species. We propose that the link between BriC, FabT, and phospholipid composition contributes to the ability of S. pneumoniae to alter membrane homeostasis in response to the production of a quorum-sensing peptide.

**IMPORTANCE** Adaptation of bacteria to their host environment is a key component of colonization and pathogenesis. As an essential component of bacterial membranes, fatty acid composition contributes to host adaptation. Similarly, cell-cell communication, which enables population level responses, also contributes to host adaptation. While much is known about the pathways that control the biosynthesis of fatty acids, many questions remain regarding regulation of these pathways and consequently the factors that affect the balance between unsaturated and saturated fatty acids. We find that BriC, a cell-cell communication peptide implicated in biofilm regulation and colonization, both is influenced by a fatty acid biosynthesis pathway and affects this same pathway. This study identifies a link between cell-cell communication, fatty acid composition, and biofilms and, in doing so, suggests that these pathways are integrated into the networks that control pneumococcal colonization and host adaptation.

## INTRODUCTION

Streptococcus pneumoniae (pneumococcus) is a Gram-positive bacteria with high pathogenic potential. Worldwide, it is responsible for over one million annual deaths in children and the elderly (1). Drug-resistant pneumococcus is classified as a serious threat by the CDC, highlighting a need for new therapies. Fatty acid synthesis is a core function of the bacterial cell and, as such, serves as a potential drug target.

In bacteria, fatty acids can be acquired by two independent pathways: *de novo* production or uptake from host cells. *De novo* phospholipid synthesis in pneumococcus is carried out by 13 dissociated fatty acid synthesis genes that are part of the FASII system. These proteins are encoded in a single cluster on the genome and act to elongate and modify acetyl-CoA primers to produce saturated and unsaturated acyl chains attached to an acyl carrier protein (ACP). Attachment to ACP allows binding of any acyl chain to FASII enzymes. Uptake from the host is mediated by the fatty acid kinase (Fak) system that incorporates exogenous fatty acids into the phospholipid membrane (2, 3).

In pneumococcus, the regulation of the FASII locus is under the control of FabT, which autoregulates itself and represses most FASII genes except for *fabM* (4, 5). At basal levels, FabT binds with low affinity to the promoter regions of *fabT* and *fabK*, thereby permitting some transcription of genes in the FASII locus. Long-chained acyl-ACPs, from FASII or exogenous sources, regulate FASII through FabT. Specifically, the FabT-acyl-ACP complex strengthens FabT’s affinity for DNA and blocks FASII gene transcription (2, 3, 6).

In the current model, the balance between unsaturated (UFA) and saturated fatty acids (SFA) requires an isomerase, FabM. FabM catalyzes the conversion of *trans*-2- to *cis*-3-enoyl-ACP (4, 5, 7). The UFA:SFA ratio is determined by the competition of FabM and FabK for the available enoyl-ACP. If FabK utilizes the enoyl-ACP, SFA are produced, and if FabM utilizes the intermediate, UFA are produced. Because FabM catalyzes an equilibrium reaction, its overexpression has little effect on UFA levels compared to the larger effects of independently manipulating either the FabK or FabF levels (4). Because FabT represses FabK and FabF, but not FabM, modulation of FabT repression levels by the combination of *fabT* expression and/or acyl-ACP levels affects the balance between SFA and UFA synthesis.

In many Gram-positive bacteria, including pneumococcus, uptake of fatty acids blocks *de novo* synthesis by triggering an inhibition of endogenous fatty acid synthesis (via FabT in the pneumococcus). For example, like S. pneumoniae, Enterococcus faecalis carries two *acp* genes: *acpA* carried within the fatty acid synthesis (*fab*) operon and *acpB* carried in an operon with the acyl-ACP:phosphate transacylase *plsX*. Long-chain acyl-ACP-dependent repression via exogenous fatty acids is selective for AcpB in E. faecalis. The transcription of two ACPs, present in different neighborhoods, ensures that acyl-ACPs originating from a host will regulate FASII synthesis; incoming acyl chains are paired with AcpB while *acpA* and *fabT* are repressed (8). In this manner, many bacteria, via their ability to synthesize membrane from fatty acids acquired from the host, can survive without *de novo* synthesis as long as external sources are available. Another factor that regulates FASII is the WalRK histidine kinase signal transduction system (also known as YycFG and VicRK). Overexpression of the response regulator, WalR, modifies the expression of 12 FAS genes and results in cells phenotypically similar to *fabT* mutants that have longer-chained fatty acids (9).

Immediately downstream of the pneumococcal FASII locus is the small peptide BriC (biofilm regulator induced by competence). BriC is a ribosomally synthesized peptide that belongs to the class of double-glycine secreted peptides in pneumococcus (10). The expression of *briC* is induced directly by ComE, the master regulator of competence, and BriC is secreted via the competence-associated ABC transporter, ComAB (11). Some pneumococcal isolates, including those from the clinically important PMEN1 and PMEN14 lineages, encode a RUPB1-containing *briC* promoter which provides a competence-independent induction of *briC* in an otherwise competence-dependent pathway. The production and secretion of BriC promote late-stage biofilm development *in vitro* and nasopharyngeal colonization in a murine model of pneumococcal carriage (11).

Here, we show that *briC* is cotranscribed with genes of the *fab* gene cluster and that its expression modulates the membrane fatty acid composition of S. pneumoniae. In accordance with the role of BriC in promoting biofilm development, decreasing levels of *briC* by decoupling its transcription from the *fab* gene cluster negatively influences biofilm development. The *briC* knockout strains have reduced levels of *fabT* expression coupled with a distinct shift in membrane phospholipid molecular species composition. Thus, BriC contributes to the regulation of S. pneumoniae FASII either directly or indirectly by altering the transcription of the FabT regulon.

## RESULTS

### *briC* is cotranscribed with genes of the *fab* gene cluster.

**FIG 1:**
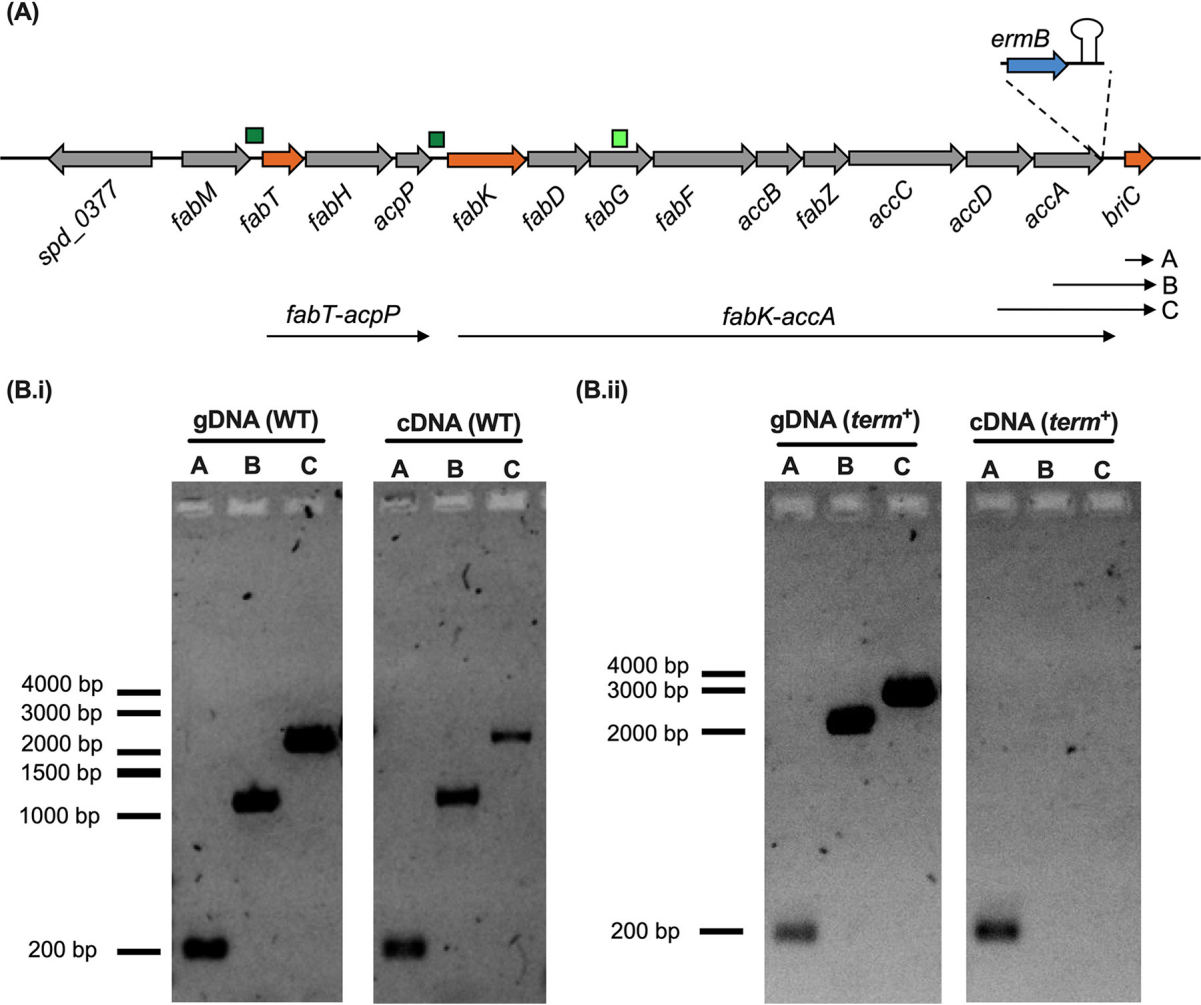
*briC* is cotranscribed with genes of the *fab* gene cluster. (A) Genomic organization of the *fab* gene cluster and *briC*. The *fab* gene cluster consists of 13 genes ranging from *fabM* to *accA*. FabT regulates the expression of two operons: *fabT*-*acpP* and *fabK*-*accA*. *briC* is situated downstream of *accA*. Small, dark green boxes indicate promoters with a FabT-binding site, while the light green box indicates an additional putative promoter. Inset: the *term*^+^ strain contains *ermB* cassette followed by the terminator B1002 immediately downstream of *accA*. Transcripts *fabT*-*acpP* and *fabK*-*accA* are labeled. Labels A, B, and C refer to the different transcripts tested below. (B.i) *briC* is transcriptionally linked to genes of the *fab* gene cluster. gDNA and cDNA from WT strain were amplified using primers expected to produce three different amplicons, visualized on agarose gel: A, *briC* only (177 bp); B, *accA*-*briC* (1,088 bp); and C, *accD*-*briC* (1,912 bp). (B.ii) Insertion of the terminator relieves cotranscription of *briC* with *accA*. gDNA and cDNA from *term*^+^ strain were amplified using primers expected to produce amplicons A, B, and C (as above), visualized on agarose gel. cDNA for *term*^+^ shows a positive band only for *briC* alone. Amplicons B and C from gDNA have a higher molecular size in *term*^+^ than in WT because of the presence of the *ermB* cassette and terminator (additional 1,051 bp).

BriC is a competence-induced gene product, yet a basal level of transcription is observed even in the absence of competence (11). The coding region for *briC* is immediately downstream of the *fab* gene cluster (159 bp downstream of *accA* [*spd_0390*] in strain R6D) (Fig. 1A). Thus, we hypothesized that the basal levels of *briC* may be attributed to its cotranscription with genes of the *fab* gene cluster. Since the genes spanning from *fabK* through *accA* are transcribed as a polycistronic unit (4), we tested whether *briC* is transcribed with the last two genes of this operon: *accD* and *accA*. We performed PCR on cDNA synthesized using RNA from planktonic pneumococcal cultures as a template and used it to determine whether transcripts extend from *accD* or *accA* to *briC* (Fig. 1B.i). The results indicated that *briC* is cotranscribed with genes of the *fab* gene cluster. An *in silico* search reveals two putative promoter sequences, which may drive the cotranscription of *briC* and genes of the *fab* gene cluster (Fig. 1A). The first is upstream of *fabK*, and the second is within the coding sequence of *fabG*. These promoters contain putative −35 and −10 regions and are in agreement with promoters previously identified by Cappable-seq (12).

To study the importance of coregulation of *briC* with genes of the *fab* gene cluster, we opted to decouple the transcription of *briC* from that of the *fab* gene cluster. We generated a strain with a transcriptional terminator immediately downstream of *accA* (*term*^+^ strain). Addition of an antibiotic selection cassette and a terminator downstream of *accA* did not affect the stability of *fabK-accA* transcript (*fabK* and *accA* are expressed 0.93- and 1.57-fold, respectively, in *term*^+^ strain relative to the wild type [WT]). As expected, while a transcript with *briC* alone is present in the *term*^+^ strain, the *accA-briC* transcript is no longer detected (Fig. 1B.ii). Thus, introduction of the terminator relieved *briC* of its cotranscription with genes of the *fab* gene cluster. Further, the competence-dependent induction of *briC* was preserved in the *term*^+^ strain (*briC* was induced 3.26-fold following treatment with Competence stimulating peptide [CSP]). We conclude that *briC* expression can be regulated in concurrence with the *fab* gene cluster, as well as independently via CSP.

### Coexpression of *briC* with genes from the *fab* gene cluster contributes to biofilm development.

**FIG 2:**
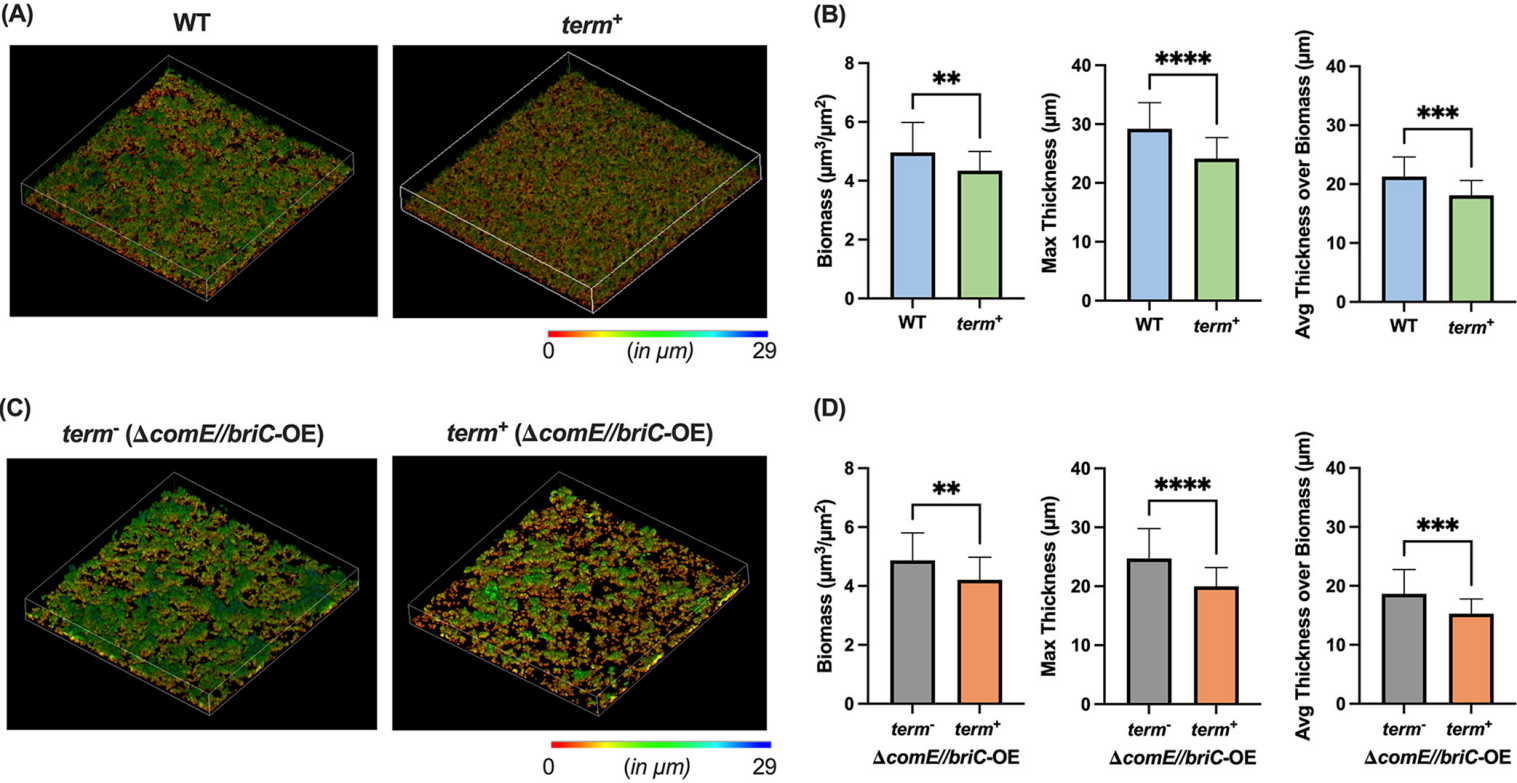
Coexpression of *briC* with the *fab* gene cluster promotes biofilm development. (A and C) Representative confocal microscopy images showing top view of the reconstructed biofilm stacks of *term*^−^ and *term*^+^ cells in strain R6D stained with SYTO59 dye at 72 h in (A) a WT and (C) a Δ*comE*//*briC*-OE genomic background. Images are pseudocolored according to depth (scales shown). (B and D) COMSTAT2 quantification of 72 h biofilm images. *y* axis denotes units of measurement: μm^3^/μm^2^ for biomass and μm for maximum thickness and average thickness over biomass. Error bars represent standard error of the mean calculated for biological replicates (*n* = 3); **, *P < *0.01; ***, *P < *0.001; and ****, *P < *0.0001 using Student’s *t* test.

We have previously demonstrated that BriC promotes biofilm development. Specifically, we demonstrated that deletion of *briC* leads to a 35% reduction in biofilm thickness and that overexpression of *briC* can rescue a biofilm defect caused by deletion of *comE* (11). Since *briC* can be cotranscribed with the upstream fatty acid genes, we hypothesized that decoupling *briC* from fatty acid synthesis would influence levels of *briC* and negatively affect biofilm development. In support, we observe an approximately 15% reduction in biofilm biomass and thickness in the *term*^+^ cells relative to those in WT cells when testing biofilms at 72 h postseeding on abiotic surfaces (Fig. 2A and B). While maximum thickness is a measure of the distance of highest point or the peak from the bottom layer containing biomass, the average thickness over biomass is an indicator of the general shape and spatial size of the biofilm.

To establish whether the biofilm defect was associated with an alteration in the competence-dependent induction of *briC*, we tested biofilm development in cells where *briC* was regulated in a competence-independent fashion. In our previous work, we generated a *briC* overexpressor strain where a second copy of *briC* is inserted into the genome; its transcription is controlled by a promoter that contains a RUPB1 sequence that results in overexpression of *briC* in a competence-independent manner (P*briC*_long_*-briC*, referred to as *briC-*OE for simplicity) (11). Strains with *comE*-deletion display a reduction in biofilm biomass and thickness relative to those of the wild type, yet RUP-driven overexpression of *briC* in this *comE*-deletion background rescues this biofilm defect (11). Thus, we tested whether decoupling transcription of *briC* from the *fab* gene cluster, by the introduction of terminator (*term*^+^), influences biofilms in this *comE*-deletion mutant (referred to as Δ*comE*//*briC*-OE). Akin to the WT background, presence of the terminator (*term*^+^) leads to a significant reduction in biomass and thickness of biofilms in the Δ*comE*//*briC*-OE strain compared to those with the *term*^−^ strain in the same background (Fig. 2C and D). These results strongly suggest that induction of *briC* via control of the *fab* gene cluster contributes to the role of BriC in promoting biofilm development. Thus, regardless of the mechanism of induction, increased expression of *briC* positively contributes to biofilm development.

### BriC contributes to membrane compositional homeostasis.

**FIG 3:**
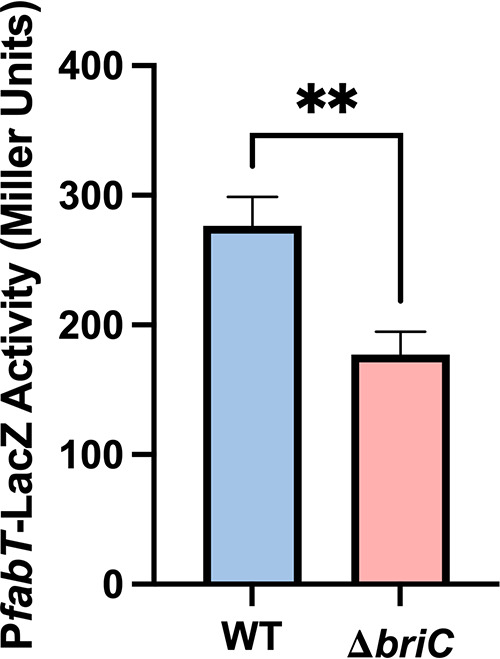
BriC induces the levels of *fabT*. β-Galactosidase assay comparing the LacZ activity of *fabT* promoter in WT and Δ*briC* cells. Cells were grown in TY medium until mid-log phase. *y* axis denotes promoter activity in Miller units expressed in nmol *p*-nitrophenol/min/ml. Error bars represent standard error of the mean for biological replicates (*n* = 3); “ns” denotes statistically nonsignificant comparison; **, *P < *0.01 using Student’s *t* test.

BriC is a secreted peptide that is cotranscribed with genes of the *fab* gene cluster. Owing to this genomic organization, we investigated whether BriC played any functional role in altering fatty acid synthesis in pneumococcal cells. FabT regulates genes of the *fab* gene cluster, including itself (4). To test the impact of BriC on expression of *fabT*, we generated a fusion of the *fabT* promoter with *lacZ* and measured the β-galactosidase activity in WT and Δ*briC* strains. We observed an approximately 35% decrease in β-galactosidase activity in Δ*briC* relative to that in WT cells (Fig. 3). We conclude that BriC enhances *fabT* transcription from its autoregulated promoter. The reduced expression of *fabT* signals repression of the entire regulon, suggesting that the absence of *briC* expression would alter the membrane phospholipid composition. Specifically, the repression of the FabT regulon would increase UFA biosynthesis at the expense of SFA (4).

This prediction was tested by determining the composition of the membrane phospholipids in wild-type and Δ*briC* strains. Pneumococcus uses the FASII system to produce acyl chains which are transferred, via positionally specific acyltransferases, to the 1 and 2 positions of glycerol-3-phosphate (G3P) that determine the composition of phosphatidic acid, the precursor to all membrane glycerolipids. We employed liquid chromatography-mass spectrometry (LC-MS) to determine the phosphatidylglycerol (PG) membrane molecular species composition in wild-type, Δ*briC*, and *briC*-overexpressing cells (*briC*-OE) cells. Liquid chromatography-tandem mass spectrometry (LC-MS) analysis determines the total carbon number of acyl chains in the 1 and 2 positions of the G3P backbone, as well as the number of double bonds, and is an accurate representation of the acyl chain production of pneumococcal strains.

**FIG 4:**
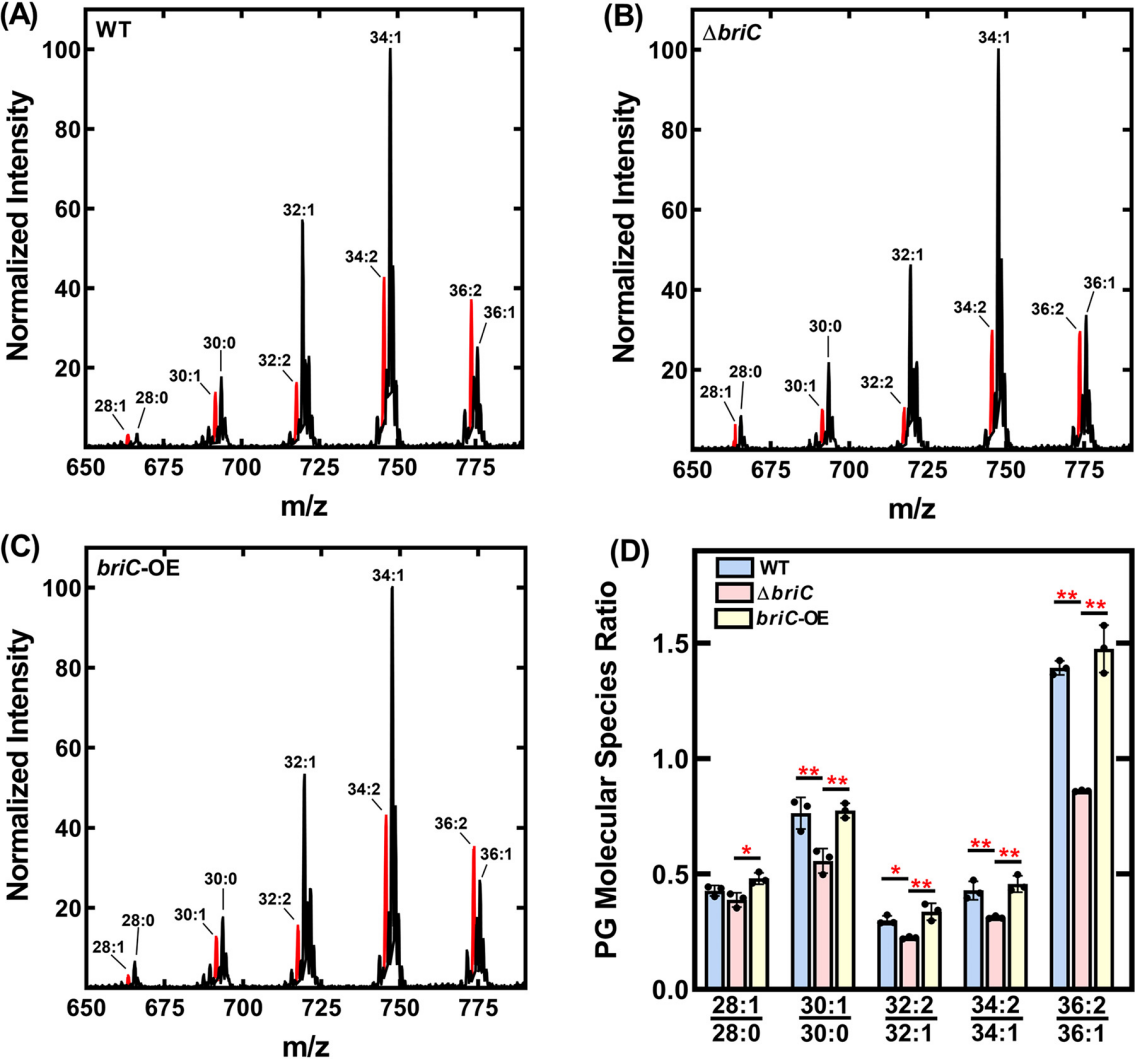
BriC promotes a membrane composition enriched in unsaturated fatty acids. Mass spectrometry analysis of the PG molecular species of R6D pneumococcal strain and its isogenic mutants. Representative spectrum of PG molecular species of (A) WT, (B) Δ*briC*, and (C) *briC*-OE strains. The most unsaturated molecular species for each carbon number is highlighted in red. (D) The PG species from three biological replicates were quantified and the ratios for each carbon number were calculated. *P* values were calculated in Prism software using ANOVA followed by Tukey’s test for multiple comparisons.

**FIG 5:**
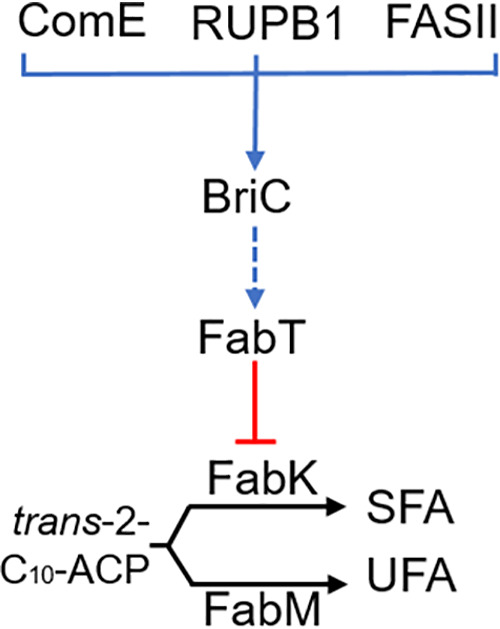
Model for BriC regulation of the FASII branch point in unsaturated fatty acid synthesis. The expression of *briC* is supported by either ComE, the RUPB1 enhancer, or cotranscription with the FabT regulon. Signaling through BriC results in the upregulation of *fabT* expression (this may be direct or indirect and is denoted with a dashed arrow). FabT is a transcriptional repressor of *fabK*, but not *fabM*, altering the branch point in FASII where FabK and FabM compete for the *trans-2*-decenoyl(C_10_)-ACP intermediate (4). NADH-dependent FabK reduction produces a saturated acyl-ACP, whereas FabM isomerization creates an unsaturated acyl-ACP. Both intermediates are elongated by FabF to become saturated (SFA) and unsaturated (UFA) fatty acids, respectively. Tilting the competitive balance between FabK and FabM alters the UFA:SFA ratio.

The wild-type strain made primarily mono- and di-unsaturated PG molecular species with the predominant peaks containing 32, 34, or 36 carbons (Fig. 4A). The molecular species distribution in these samples consisted of 16:0, 16:1, 18:0, and 18:1 acyl chains and is typical of other pneumococcal strains (2, 13). The acyl chains comprising the predominant peaks correspond to 16:0/16:1 (32 carbons), 18:1Δ11/16:1 and 16:0/18:1Δ11 (34 carbons), and 18:1Δ11/18:1Δ11 (36 carbons) (2). In the Δ*briC* strain, we observed a reduction in the unsaturated molecular species at each carbon number in comparison to those in the wild-type strain (Fig. 4B). None of these changes were observed in the *briC*-OE cells relative to the WT strain (Fig. 4C). The quantification of three replicates shows a consistent decrease in the unsaturated PG molecular species at each carbon number in the Δ*briC* strain (Fig. 4D). We conclude that FASII of the Δ*briC* strain produces a smaller amount of unsaturated fatty acids that, in turn, alters the membrane phospholipid molecular species composition. Together, these results suggest that BriC, via *fabT* levels, alters the lipid composition of pneumococcal cell membranes at the branch point in unsaturated fatty acid synthesis, tilting the balance toward unsaturated fatty acids (Fig. 5).

## DISCUSSION

Long-chain fatty acids serve as essential components of bacterial membranes, and compositional changes therein are essential for cellular survival and environmental adaptation. The biosynthesis of fatty acids is tightly regulated. While much is known about the synthesis of membrane lipids, gaps remain regarding the nature of the molecular signals that regulate this pathway. Here, we present evidence that BriC, a small, secreted peptide implicated in cell-cell communication, is cotranscribed with genes of the *fab* gene cluster and participates in regulation of phospholipid membrane composition via a role in *fabT* induction (Fig. 5). Moreover, as we have previously shown that BriC promotes biofilms, we demonstrate that this phenotype is linked to coregulation between FASII genes and *briC.*

The demonstration that *briC* is cotranscribed with the *fab* gene cluster reveals a third pathway for *briC* regulation (Fig. 5). The regulation of *briC* by ComE, via the ComE-binding box, is conserved across strains in the species and demonstrates a tight link between competence induction and *briC* expression (11). In a subset of strains, the transcription of *briC* is also influenced by the presence of transposable RUP (repeat unit of pneumococcus) sequence in its promoter (11). RUP allows for a CSP-independent pathway for the expression of *briC*. The discovery that *briC* can be cotranscribed with the *fab* gene cluster reveals yet another pathway for regulation. Further, it suggests that BriC may be regulated by multiple two-component systems, ComE and WalRK. WalRK triggers the activation of the *fab* gene cluster, and since this work reveals that *briC* is cotranscribed with genes of this locus, it is likely that WalRK promotes *briC* expression. The activation of *briC* by multiple pathways is consistent with a gene network where BriC is positioned to respond to diverse regulatory inputs. Signaling through WalRK is important in maintenance of cell shape, division, and pathogenesis and in the response to stresses such as oxidative stress (14–16). Competence activation has also been described as a general SOS response pathway, as well as a sensor of cell density. Thus, the colonization factor BriC may respond to cell density and stress conditions and induce changes in membrane composition and biofilm growth.

BriC is a double-glycine peptide exported from cell via ComAB (11). ComAB-mediated processing and export of BriC are critical for rescuing biofilm defects of the cell. Decoupling *briC* from the FASII locus leads to a decrease in biofilm robustness. Thus, we propose that the influence of *briC* on membrane composition requires an active secreted form of BriC.

We have previously demonstrated that BriC promotes biofilm development and nasopharyngeal colonization (11). In this study, we show that BriC influences membrane lipid composition. Are these phenotypes connected? A study comparing transcriptional profiles of pneumococcal cells growing in biofilm versus in planktonic mode of growth found an upregulation of fatty acid biosynthesis genes during biofilm development (17). A role for fatty acid biosynthesis and metabolism in biofilm formation has also been reported in other bacteria, including Bacillus subtilis, Staphylococcus aureus, and Pseudomonas aeruginosa (18–21). It seems plausible that BriC-dependent changes in membrane properties contribute to biofilm development. Alternatively, enhanced cell-cell signaling associated with a biofilm-mode of growth may enhance BriC-mediated effects on lipid composition and serve as a link between biofilms and lipid composition.

In this work, we have revealed a link between a cell-cell communication peptide and the regulation of fatty acid composition. Our findings reveal that *briC* is coregulated with the *fab* gene cluster and, reciprocally, that it affects membrane homeostasis by influencing transcription of the FabT regulon.

## MATERIALS AND METHODS

### Bacterial strains and growth conditions.

The experimental work was performed with the R6D wild-type strain of Streptococcus pneumoniae (Hun663.tr4), as this was used in our previous studies of BriC (11). Colonies were grown from frozen stocks by streaking on TSA-II agar plates supplemented with 5% sheep blood (BD BBL, NJ, USA). Unless otherwise stated, streaked colonies were picked and inoculated in fresh Columbia broth (Remel Microbiology Products, Thermo Fisher Scientific, USA) whose pH was adjusted to 6.6 by the addition of 1 M HCl and thereafter incubated at 37°C and 5% CO_2_ without shaking. Antibiotics were not added to the growth medium under any assay conditions.

### Construction of mutants.

Mutant strains were constructed by using site-directed homologous recombination and selected by the addition of an antibiotic resistance marker. The *term*^+^ transformation construct was generated by ligating the amplified flanking regions with antibiotic resistance cassette followed by transcriptional terminator B1002. Between 1 to 2 kb of flanking regions upstream and downstream of the region of interest were amplified from parental strain using Q5 2× master mix (New England Biolabs, USA). The antibiotic resistance gene *ermB* and its promoter were amplified from S. pneumoniae SV35-T23. The sequence of the terminator was added to the primers. The PCR products were assembled by Gibson assembly using NEBuilder HiFi DNA assembly cloning kit (New England Biolabs, USA). The promoter, *ermB*, and terminator were inserted downstream of *accA* decoupling the transcription of *briC* from FASII locus.

### Bacterial transformations.

Bacterial target strains were grown in acidic Columbia broth until they reached an optical density at 600 nm (OD_600_) of 0.05 and followed by addition of 125 μg/ml of CSP1 (sequence: EMRLSKFFRDFILQRKK; purchased from GenScript, NJ, USA) and 1 μg of transforming DNA. The cultures were incubated at 37°C and 5% CO_2_ without shaking for 2 h followed by plating on Columbia agar plates containing the appropriate antibiotic, kanamycin (150 μg/ml) or erythromycin (2 μg/ml), and incubating overnight. Resistant colonies were cultured in selective media, and the colonies were confirmed using PCR. Bacterial strains generated in this study are listed in Table S1.

10.1128/mSphere.00145-21.1 TABLE S1 Strains used in this experimental work. Download Table S1, XLSX file, 0.01 MB. Copyright © 2021 Aggarwal et al. 2021 Aggarwal et al. https://creativecommons.org/licenses/by/4.0/ This content is distributed under the terms of the Creative Commons Attribution 4.0 International license.

### RNA extractions.

For quantitative reverse transcription PCR (qRT-PCR), samples were grown in acidic Columbia broth until they reached an OD_600_ of 0.3 to 0.4. This was followed by addition of RNALater to preserve RNA quality and pelleting of cells. The cells were lysed by resuspending the pellet in an enzyme cocktail (2 mg/ml proteinase K, 10 mg/ml lysozyme, and 20 μg/ml mutanolysin). Then, RNA was isolated using the RNeasy kit (Genesee Scientific, USA) following the manufacturer’s instructions. Contaminant DNA was removed by treating with DNase (2 units/μl) at 37°C for at least 45 min followed by RNA purification using the RNeasy kit. The RNA concentration was measured by NanoDrop 2000 spectrophotometer (Thermo Fisher Scientific, USA). The purity of the RNA samples was confirmed by the absence of a DNA band on an agarose gel obtained upon running PCR products for the samples amplified for *gapdh*.

### qRT-PCR.

Purified RNA (500 ng) was used as a template for first-strand cDNA synthesis by using qScript cDNA synthesis kit (Quantabio, USA) followed by qRT-PCR using PerfeCTa SYBR green SuperMix (Quantabio, USA) in an Applied Biosystems 7300 instrument (Applied Biosystems, USA). Geometric mean of *gyrB* and 16S rRNA counts were used for normalization.

### Biofilm development assay.

For biofilm development assays, pneumococcal cells were grown in acidic Columbia broth until the cultures reached an OD_600_ of 0.05. Antibiotics were not added to the media. Then, 3 ml of culture was seeded on 35-mm glass bottom culture dishes (MatTek Corporation, USA) and incubated at 37°C and 5% CO_2_ without shaking. At 24 h and 48 h postseeding, the supernatant from the dishes was carefully aspirated with a pipette, followed by the addition of the same volume of prewarmed medium made at one-fifth of the original concentration. The biofilms were fixed for analysis at 72 h postseeding. For fixation, supernatants were aspirated and the biofilms were washed thrice with phosphate-buffered saline (PBS) to remove nonadherent and weakly adherent cells. Thereafter, biofilms were fixed with 4% paraformaldehyde (Electron Microscopy Sciences, USA) for 20 min. The biofilms were then washed with PBS three times and stained for confocal microscopy.

### Confocal microscopy and quantification of biofilms.

SYTO59 nucleic acid stain (Life Technologies, USA) was used to stain biofilms as per the manufacturer’s instructions for 30 min. The stained biofilms were then washed three times and preserved in PBS for imaging. Imaging was performed on the stage of Carl Zeiss LSM-880 META FCS confocal microscope using 561 nm laser for SYTO dye. Z-stacks were captures at every 0.46 μm, imaged from the bottom to the top of the stack until cells were visible, and reconstructed in Carl Zeiss black edition and ImageJ. The biofilm stacks were analyzed using COMSTAT2 plug-in for ImageJ (22), and the different biofilm parameters (biomass, maximum thickness, and average thickness over biomass) were quantified. For depiction of representative reconstructed Z-stacks, empty slices were added to the images so the total number of slices across all the samples were the same. The reconstructed stacks were pseudocolored according to depth using Carl Zeiss black edition. Each biofilm experiment was performed at least three independent times, and each biological replicate contained two technical replicates. For each technical replicate, images were recorded at multiple different locations on the dishes.

### Construction of *lacZ* fusions.

Chromosomal transcriptional *lacZ* fusions to the target promoters were constructed as previously described (11). Briefly, *lacZ* fusions were generated in the *bgaA* gene using modified integration plasmid pPP2. The *fabT* promoter region was amplified from R6D strains and modified to contain KpnI and XbaI restriction sites. The products were then digested with restriction enzymes followed by sticky-end ligation of the products. These plasmids were transformed into Escherichia coli TOP10 strain and selected on LB (Miller’s modification, Alfa Aesar, USA) plates, supplemented with ampicillin (100 μg/ml). The plasmids were then purified by using E.Z.N.A. plasmid DNA minikit II (OMEGA bio-tek, USA), transformed into pneumococcal strains, and selected on Columbia agar plates supplemented with kanamycin (150 μg/ml).

### β-Galactosidase assay.

β-galactosidase assay was performed as previously described (23). For assaying the β-galactosidase activity, cells were grown in TY medium (TH medium supplemented with 0.5% yeast extract) until exponential phase, pelleted, and frozen. The frozen cells were thawed and reinoculated in TY medium and grown until midexponential phase for assaying the activity.

### Membrane lipid composition analysis.

Bacterial cells were inoculated in chemically defined medium (CDM) glucose and incubated at 37°C and 5% CO_2_ without shaking until they reached an OD_600_ of 0.5. CDM-glucose was prepared as previously described (24). The cells were then pelleted by centrifuging at 4,000 rpm for 15 min followed by washing with PBS three times. The PBS was decanted and the washed cells were frozen at −20°C before being resuspended in 1 ml deionized water and vortexed. Lipids were resuspended in chloroform:methanol (2:1) and extracted using the Bligh and Dyer method (25). PG was analyzed using a Shimadzu Prominence UFLC attached to a QTrap 4500 equipped with a Turbo V ion source (Sciex). Samples were injected onto an Acquity UPLC BEH HILIC, 1.7 μm, 2.1 × 150 mm column (Waters) at 45°C with a flow rate of 0.2 ml/min. Solvent A was acetonitrile, and solvent B was 15 mM ammonium formate, pH 3. The high-pressure liquid chromatography (HPLC) program was the following: starting solvent mixture of 96% A/4% B, 0 to 2 min isocratic with 4% B, 2 to 20 min linear gradient to 80% B, 20 to 23 min isocratic with 80% B, 23 to 25 min linear gradient to 4% B, and 25 to 30 min isocratic with 4% B. The QTrap 4500 was operated in the Q1 negative mode. The ion source parameters for Q1 were as follows: ion spray voltage −4,500 V, curtain gas 25 lb/in^2^, temperature 350°C, ion source gas 1 40 lb/in^2^, ion source gas 2 60 lb/in^2^, and declustering potential −40 V. The system was controlled by the Analyst software (Sciex). The sum of the areas under each peak in the mass spectra was calculated, and the percentage of each molecular species present was calculated with LipidView software (Sciex).

### Statistical tests.

For comparisons between only two groups, Student’s *t* test was performed. *P* values of less than 0.05 were considered to be statistically significant. Statistical analyses of the ratios of PG molecular species were determined using an analysis of variance (ANOVA) and Tukey’s test.

10.1128/mSphere.00145-21.2 TABLE S2 Primers used in this study. Download Table S2, XLSX file, 0.01 MB. Copyright © 2021 Aggarwal et al. 2021 Aggarwal et al. https://creativecommons.org/licenses/by/4.0/ This content is distributed under the terms of the Creative Commons Attribution 4.0 International license.
